# 

**Published:** 1806-09

**Authors:** 


### LIST OF NEW MEDICAL PUBLICATIONS.

A Treatise on Vaccine Inoculation; to which is added, an Account of the Chicken Pox, and the Hives; with an Appendix, containing Letters from Physicians and Surgeons of eminence respecting the present State of Vaccination in many Cities and principal Towns of the United Kingdom. By Robert Willan, M. D. F. A. S. &c. &c. 4to. 15s. boards.

A Letter to the Editor of the British Critic, occasioned by some Remarks in that Review on a Book intitled "Cases of Pulmonary Consumptions, and treated with Uva Ursi," by the Author. 1s.

---

### *TO CORRESPONDENTS.*

Mr. Fowler, of Melton Mowbray, wishes to learn where he may find the Report of the Case, in which the worm lozenges produced excessive salivation, mortification, and death.

